# Development of dopaminergic genetic associations with visuospatial, verbal and social working memory

**DOI:** 10.1111/desc.12889

**Published:** 2019-08-06

**Authors:** Iroise Dumontheil, Emma J. Kilford, Sarah‐Jayne Blakemore

**Affiliations:** ^1^ Department of Psychological Sciences, Centre for Brain and Cognitive Development Birkbeck, University of London London UK; ^2^ Institute of Cognitive Neuroscience University College London London UK

**Keywords:** COMT, development, dopamine, genetic, social cognition, working memory

## Abstract

Dopamine transmission in the prefrontal cortex (PFC) supports working memory (WM), the temporary holding, processing and manipulation of information in one's mind. The gene coding the catechol‐*O‐*methyltransferase (COMT) enzyme, which degrades dopamine, in particular in the PFC, has a common single nucleotide polymorphism leading to two versions of the COMT enzyme which vary in their enzymatic activity. The methionine (Met) allele has been associated with higher WM performance and lower activation of the PFC in executive function tasks than the valine (Val) allele. In a previous study, *COMT* genotype was associated with performance on verbal and visuospatial WM tasks in adults, as well as with performance on a novel social WM paradigm that requires participants to maintain and manipulate information about the traits of their friends or family over a delay. Here, data collected in children and adolescents (*N* = 202) were compared to data from the adult sample (*N* = 131) to investigate possible age differences in genetic associations. Our results replicate and extend previous work showing that the pattern of superior WM performance observed in Met/Met adults emerges during development. These findings are consistent with a decrease in prefrontal dopamine levels during adolescence. Developmentally moderated genetic effects were observed for both visuospatial and social WM, even when controlling for non‐social WM performance, suggesting that the maintenance and manipulation of social information may also recruit the dopamine neurotransmitter system. These findings show that development should be considered when trying to understand the impact of genetic polymorphisms on cognitive function.

## Graphical abstract

Rs4680 is a genetic polymorphism with two allelic variants (Met vs. Val) that affects the enzymatic activity of the catechol‐*O‐*methyltransferase (COMT) enzyme, which degrades dopamine. Met/Met adults have been found to have better performance and lower prefrontal cortex activity during working memory (WM) tasks. This study shows that the genetic association with visuospatial WM, but not verbal WM performance, emerges during development, and that the same pattern can be observed in a WM task requiring participants to manipulate social information. These results are consistent with a decrease in prefrontal dopamine levels during development and suggest an involvement of dopaminergic neurotransmission in social cognition.

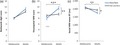

**Research Highlights**

- A group of 220 children and adolescents were compared to a previously studied sample of 131 adults on three working memory (WM) tasks.
- Participants were genotyped for the common rs4680 variant of gene coding the catechol‐*O‐*methyltransferase (COMT) enzyme, which affects prefrontal dopamine transmission.
- The association between *COMT* genotype and visuospatial WM performance emerged during adolescence, replicating previous findings, a pattern consistent with decreasing prefrontal dopamine levels during development.
- We further observed that the association between social WM and *COMT* genotype also changes during development, demonstrating a possible involvement of dopamine neurotransmission in social cognition.

## 1 INTRODUCTION

Working memory (WM) refers to the temporary holding, processing and manipulation of information in one's mind. Dopamine transmission in the prefrontal cortex (PFC) is critically involved in WM, as evidenced from electrophysiological and pharmacological studies in animals (Brozoski, Brown, Rosvold, & Goldman, 1979; Levy & Goldman‐Rakic, 2000) and neuroimaging (Cropley, Fujita, Innis, & Nathan, 2006; Fischer et al., 2010; McNab et al., 2009; Ziermans et al., 2012) and pharmacological studies in humans (e.g. Mehta et al., 2000; Müller, von Cramon, & Pollmann, 1998). This research has led to the suggestion that prefrontal dopamine facilitates the stabilization of information in WM (Cools & D’Esposito, 2011; de Frias et al., 2010), and that PFC functioning and WM performance follow an inverted U‐shaped function, whereby both deficient and excessive amounts of prefrontal dopamine activity predict poor task performance (Arnsten, 1997; Cools & D’Esposito, 2011; Vijayraghavan, Wang, Birnbaum, Williams, & Arnsten, 2007).

Another approach to study the role of neurotransmitter systems in cognition is through the study of common genetic polymorphisms. The gene coding for the catechol‐*O‐*methyltransferase (COMT) enzyme, which mediates the degradation of catecholamines, in particular dopamine, has been a gene of particular interest in the study of WM and executive functions more broadly. The rs4680 Valine158Methionine (Val^158^Met) single nucleotide polymorphism (SNP) leads to a reduction in COMT enzymatic activity in Met carriers (Chen et al., 2004; Männistö & Kaakkola, 1999; Weinshilboum, 2006). The Met allele has been shown to be associated with superior WM performance (Diaz‐Asper et al., 2008; Dumontheil et al., 2011; Goldberg et al., 2003), and reduced PFC activation has been observed during executive function tasks (Dickinson & Elvevåg, 2009; de Frias et al., 2010; Mier, Kirsch, & Meyer‐Lindenberg, 2010; Tunbridge, Harrison, & Weinberger, 2006; Witte & Flöel, 2012), although no meta‐analytic support was found for these neuroimaging findings (Nickl‐Jockschat, Janouschek, Eickhoff, & Eickhoff, 2015). Interestingly, pharmacologically increasing dopamine concentrations by administrating amphetamine (which increases dopamine release), or tolcapone (a COMT inhibitor) to healthy adults leads to worsened WM performance and increased PFC activation in Met/Met individuals, while performance improves in Val/Val individuals (Giakoumaki, Roussos, & Bitsios, 2008; Mattay et al., 2003). These pharmacological manipulation studies suggest that Met/Met individuals have dopamine levels enabling optimal PFC functioning, while the lower dopamine levels of Val/Val individuals may be suboptimal (Meyer‐Lindenberg & Weinberger, 2006). However, the association between rs4680 and WM performance has not always been consistently observed (e.g. Blanchard, Chamberlain, Roiser, Robbins, & Müller, 2011) and depends on the population studied and the specific paradigm used (see Barnett, Scoriels, & Munafò, 2008, for meta‐analysis, and Dickinson & Elvevåg, 2009; Witte & Flöel, 2012, for reviews).

Research in the past has typically distinguished between verbal and visuospatial WM, referring to the nature of the information being maintained. Social WM is the ability to store and manipulate information about other people (Meyer, Spunt, Berkman, Taylor, & Lieberman, 2012; Meyer, Taylor, & Lieberman, 2015). While verbal and visuospatial WM tasks are associated with increased activation in the lateral fronto‐parietal cortex (Owen, McMillan, Laird, & Bullmore, 2005; Rottschy et al., 2012; Van Overwalle, 2009), social cognition tasks, in particular those requiring the processing of one's own or other's mental states (mentalizing) are associated with increased activation in the medial PFC, temporal cortex, and precuneus or posterior cingulate cortex (Van Overwalle, 2009), a network of brain regions often referred to as the “social brain” (Frith & Frith, 2010). Using neuroimaging, Meyer and colleagues (Meyer et al., 2012, 2015) have demonstrated that, during a social WM task, both the medial and lateral fronto‐parietal systems show WM load‐dependent increases in activation, suggesting that the social brain and typical WM systems work in parallel to support social WM.

The PFC undergoes prolonged structural and functional changes during adolescence (Crone & Dahl, 2012), and is associated with the continued maturation of a range of PFC‐mediated cognitive processes, including both WM and social cognition (Burnett, Sebastian, Cohen Kadosh, & Blakemore, 2011; Dumontheil, 2016; Luna, Padmanabhan, & O’Hearn, 2010). Behavioural and neuroimaging studies of the development of complex aspects of social cognition, such as perspective‐taking (Dumontheil, Hillebrandt, Apperly, & Blakemore, 2012; Dumontheil, Küster, Apperly, & Blakemore, 2010) and social decision‐making (Blakemore & Robbins, 2012; Burnett et al., 2011; Magis‐Weinberg, Blakemore, & Dumontheil, 2017), suggest that developments in executive functions and social cognition mutually influence each other. Thus, the improved integration of social cognitive and fronto‐parietal systems in adolescence may also contribute to developmental advances in other aspects of cognition that require the integration of social cognition with more domain‐general cognitive control processes, such as social WM.

Little is known of the role of dopamine in social cognition (Skuse, 2006; Skuse & Gallagher, 2011). However, we have previously shown that the rs4680 variant of *COMT* was associated with individual differences in performance of a social WM task in adults (Dumontheil et al., 2014). Importantly, the association was maintained when performance on standard verbal and visuospatial WM tasks was covaried out. These results, in parallel with the neuroimaging studies by Meyer and colleagues (Meyer et al., 2012, 2015), provide tentative evidence that the dopamine neurotransmitter system may also be involved in supporting social WM processing within the social brain.

Animal studies have suggested that there are significant changes occurring in the dopamine neurotransmitter system during development. Dopamine cell density in the rhesus PFC decreases by up to 50% from the onset of adolescence to late adulthood (Goldman‐Rakic & Brown, 1981), and basal dopamine levels, dopaminergic turnover and dopaminergic input in the PFC peak in early adolescence and decline thereafter in other animal studies (Andersen, Dumont, & Teicher, 1997; Rosenberg & Lewis, 1994, 1995; Teicher et al., 1993). Research has also suggested that there are peaks in D1 and D2 dopamine receptor expression around puberty in rats, with a decline in receptor numbers that occurs later in the PFC than in the striatum (see McCutcheon & Marinelli, 2009, for review). There are very few studies investigating developmental changes in the dopamine neurotransmitter system in humans. A post‐mortem study has shown a very early peak (age 2 years) in D1 receptor density in the striatum, with a slow decrease in density during subsequent decades (Seeman et al., 1987). Another post‐mortem study found that linear decreases with age in mRNA expression and/or protein levels of dopamine receptors D2, D4 and D5, tyrosine hydroxylase and COMT in the dorsolateral PFC were driven by early decreases in the first few months or years of life (Rothmond, Weickert, & Webster, 2012). The only differences observed in later development were increases in dopamine receptor D1, monoamine oxidase (MAO)‐A and MAO‐B protein levels between 6–12 and 14–17 years of age or adulthood, and an increase in MAO‐B mRNA expression between 14 and 17 years of age and adulthood (Rothmond et al., 2012). A positron emission tomography study showed a decrease in D1 binding potential during adolescence in the dorsolateral PFC, while no changes were observed in the ventral or dorsal striatum (Jucaite, Forssberg, Karlsson, Halldin, & Farde, 2010). Overall, these studies suggest there are changes in the dopamine neurotransmitter system during development, but that the pattern of changes is complex and does not appear to be consistent across species. It has been argued, mostly based on the animal data, that there may be a peak in dopamine availability in the human pubertal period, relative to inhibitory serotonin levels, and that this may explain adolescent specific behaviours such as heightened impulsivity and novelty‐seeking (Chambers, Taylor, & Potenza, 2003; Luna, Marek, Larsen, Tervo‐Clemmens, & Chahal, 2015; Padmanabhan & Luna, 2014; Wahlstrom, Collins, White, & Luciana, 2010; Wahlstrom, White, & Luciana, 2010). However, more research is needed in humans.

Findings consistent with changes in neurotransmitter systems during development have come from cognitive and neuroimaging studies using genetic polymorphisms of the serotonin and dopamine system. In a sample of 48 participants aged 9–19 years, activity in the amygdala and connectivity between the amygdala and the medial ventral PFC differed as a function of age and serotonin transporter genotype, with the low expressing genotype showing increasing activity and decreasing connectivity with age (Wiggins et al., 2014). In another study, Wahlstrom et al. (2007) estimated WM in 9–17 year olds using a composite score combining performance in digit and spatial forward and backward span tasks and a delayed visuospatial response task. *COMT* Val carriers showed poorer WM performance than Met homozygotes, in contrast to previous findings in adults (Diaz‐Asper et al., 2008; Goldberg et al., 2003). Dumontheil et al. (2011) later demonstrated in a longitudinal sample that the adult pattern of lower WM capacity and higher lateral PFC recruitment during a visuospatial WM task in Val carriers emerged during development, rather than being stable over childhood, adolescence and early adulthood. These data were considered to support the presence of higher levels of basal dopamine in late childhood and adolescence than in adulthood, leading to a shift of the position of the *COMT* genotypes on the inverted U‐shape function linking PFC functioning and dopamine levels (Dumontheil et al., 2011; Wahlstrom et al., 2007; Wahlstrom, Collins, et al., Wahlstrom, Collins, et al., 2010; Wahlstrom, White, et al., 2010).

The present study used genetic variation in *COMT* to further investigate the dopamine neurotransmitter system during development. Data were collected from a sample of children and adolescents aged 9–18 years old and compared to previously collected and published data from a sample of adults aged 20–39 years old (Dumontheil et al., 2014; Kilford, Dumontheil, Wood, & Blakemore, 2015; Magis‐Weinberg et al., 2017). We first aimed to replicate previous findings of an interaction between age and *COMT* genotype on the performance of a visuospatial WM task (Dumontheil et al., 2011). Second, we investigated whether this interaction was also observed in a verbal WM task, as suggested by results in a sample of 9–17 year olds (Wahlstrom et al., 2007), following up the genetic effects we previously observed in an adult sample (Dumontheil et al., 2014; Kilford et al., 2015). Finally, we investigated whether a similar pattern would be observed in a social WM task, over and above genetic effects on standard WM, as this would suggest that the influence of dopaminergic genetic variation on social WM also changes between childhood, adolescence and adulthood.

## 2 METHODS

### 2.1 Participants

We recruited 161 healthy adult participants (20–39 years old, 83 males) via University College London (UCL) volunteer databases; 218 child and adolescent participants (9–18 years old, 97 males) were recruited in schools in and around London. The study was approved by the UCL Research Ethics Committee, all adult participants gave written informed consent, while written informed consent was obtained from the parent or guardian of the child and adolescent participants and verbal assent was obtained from these participants themselves. Participants were individually tested in a quiet room either in the laboratory or in the participant's school on a battery of tests, which included the WM tasks and the vocabulary subtest of the Wechsler Abbreviated Scale of Intelligence (WASI; Wechsler, 1999). The adult data were previously analysed and published in Dumontheil et al. (2014) and Kilford et al. (2015).

#### 2.1.1 Participant exclusions

Two adolescent participants were excluded because of a diagnosis of a developmental disorder: one had Turner syndrome, the other had Asperger Syndrome. A further nine adolescents were excluded because of missing genetic data (six did not provide a saliva sample, DNA extraction failed for two, and genotyping failed for one participant), and seven adults were excluded because of failed genotyping. The frequency of many genetic variants, including *COMT*, varies considerably across populations (Palmatier, Kang, & Kidd, 1999). Global allele frequency distributions reveal that the Val allele at *COMT* is significantly more frequent in East Asian populations (including China and Japan) compared to European, African and Southwest Asian populations (Palmatier et al., 1999). Thus, East Asian populations may have a different ancestral haplotype of *COMT* (DeMille et al., 2002). East Asian participants in our sample (*n* = 6 adolescents, *n* = 22 adults) had indeed a greater frequency of the *COMT* Val allele than the other ethnicities, and were therefore excluded to make the distribution of genotypes more homogeneous. This gave a sample of 333 participants (202 adolescents, 131 adults; see Table 1), although final sample sizes were slightly smaller for individual tasks and measures because of task‐specific exclusions (described in 2.3 Behavioural assessments).

**Table 1:** Participant demographics

Age group	COMT genotype	*n* (male/female)	Age *M* (*SD*)	Verbal IQ^a^ *M* (*SD*)
Adolescents	Met/Met	49 (19/30)	13.13 (1.99)	113.7 (12.7)
	Val carriers	153 (75/78)	13.28 (2.05)	115.2 (11.8)
	All	202 (94/108)	13.24 (2.03)	114.8 (12.0)
Adults	Met/Met	38 (23/15)	25.22 (3.19)	113.6 (12.0)
	Val carriers	93 (41/52)	26.90 (4.09)	112.7 (12.9)
	All	131 (64/67)	26.41 (3.91)	112.9 (12.6)
Total	Met/Met	87 (42/45)	18.41 (6.56)	113.6 (12.3)
	Val carriers	246 (116/130)	18.43 (7.26)	114.2 (12.3)
	All	333 (158/175)	18.42 (7.07)	114.1 (12.3)

Note

The *n* was smaller for individual tasks and measures because of task‐specific exclusions.

a Four adults were missing Verbal IQ data (1 Met/Met, 3 Val carriers).

#### 2.1.2 Matching of age and genotypic groups

Effects of *COMT* genotype were investigated using the Val allele dominant genotype grouping (0: Met/Met, 1: Val carriers) that was found to show association with performance in our analyses of the adult data (Dumontheil et al., 2014), and has been shown in previous studies to be the most effective model for explaining the influence of *COMT* variance on behaviour (Barnett et al., 2008; Dumontheil et al., 2011). Effects of age were explored by comparing adolescent (age range: 9.0–18.0 years) and adult (age range: 20.3–39.4 years) participant groups, to minimize the need to match genotype and gender groups at the finer grained scale needed for continuous analyses of age effects. Additional exploratory post hoc analyses are presented in the Supplementary Materials, in which all three *COMT* genotypes are compared (Met/Met; Val/Met; Val/Val), or age is modelled as a continuous variable across the whole sample or within the adolescent sample only, to facilitate comparison with the results of other studies.

Group‐matching analyses were performed on the final sample with genetic data but without considering task‐specific exclusions (*n* = 333). One‐way ANOVAs on age data indicated that the genotype groups (Met/Met vs. Val carriers) were matched in the adolescent group (*F*(1,200) = 0.203, *p* = .653), while Val carriers were significantly older than Met/Met individuals in the adult group (*F*(1,129) = 5.120, *p* = .025; Table 1). Previous analyses of the adult sample showed that these age differences did not affect genetic association results (Dumontheil et al., 2014; Kilford et al., 2015), and therefore age was not included as a covariate. However, for completeness, we include an additional analysis in which the adult *COMT* groups were matched for age in the Supplementary Materials. Gender distribution was matched between the age groups (adolescents vs. adults; χ^2^(1) = 0.172, *p* = .678), and within each age group there was no difference in gender distribution between the genotype groups (adolescents: χ^2^(1) = 1.565, *p* = .211; adults: χ^2^(1) = 2.918, *p* = .088). The same pattern of results was observed in the smaller social WM task sample.

A 2 (age group) × 2 (genotype) ANOVA on the verbal IQ data indicated no main effect of age group (*p* = .405, η*_p_* ^2^ = 0.002), no main effect of genotype (*p* = .854, η*_p_* ^2^ < 0.001), and no significant interaction (*p* = .431, η*_p_* ^2^ = 0.002). This was also the case in the smaller social WM task sample. The age and genotype groups were therefore considered sufficiently matched and IQ was not included in further analyses.

In terms of ethnicity, 154 of the adolescents were Caucasian, 44 were not (7 Black (African or Caribbean), 26 Asian (not East Asian), 10 Mixed Asian (not East Asian) and Caucasian, two Other (not specified) and three did not provide ethnicity information). In the adult sample, 87 were Caucasians, 43 were not (13 Black (African or Caribbean), three Mixed Black and Caucasian, 21 Asian (not East Asian), two Mixed Asian (not East Asian) and Caucasian, four Other (not specified) and one did not provide ethnicity information). A chi‐square test indicated that there was a significantly greater proportion of Caucasians in the developmental (77.4%) than the adult sample (66.9%; χ^2^(1) = 4.394, *p* = .036). There was also a significantly greater proportion of Caucasians in the Met/Met group (72 vs. 13, 84.7%) than in Val carriers (169 vs. 75, 69.3%; χ^2^(1) = 7.674, *p* = .006). Analyses were therefore repeated with the inclusion of ethnicity (Caucasian vs. non‐Caucasian) as a covariate (the four participants with missing ethnicity were not included in these analyses).

### 2.2 Genetic analysis

Saliva samples were collected using OG‐500, Oragene‐DNA self‐Collection Kit, Oragene.DNA, as per protocol suggested by Oragene.DNA (DNA Genotek Inc.; http://www.dnagenotek.com/ROW/pdf/PD-BR-017.pdf). Adult DNA was extracted from saliva samples at the Department of Molecular Neuroscience at the Institute of Neurology, UCL, while child and adolescent DNA was extracted at the Molecular Psychiatry Laboratory, UCL. DNA was extracted using the OG‐L2P DNA extraction kit (DNA Genotek Inc.) as per protocol suggested by Oragene.DNA (http://www.dnagenotek.com/US/pdf/PD-PR-006.pdf).

The analysis of the SNP was carried out by AROS, University of Aarhus, Denmark. The *COMT* rs4680 SNP in exon 4 of the gene was characterized by an A/G substitution, which causes the Val^158^Met polymorphism. The SNP was determined using the TaqMan‐based genotyping technology from Applied Biosystems. Reactions and analysis were performed in a 384‐well plate format. All samples were normalized to 5 ng/µl of DNA. The reaction components for each genotyping reaction were as follows: 2.5 µl TaqMan master mix, 0.25 µl TaqMan assay X20, 1.25 µl water resulting in a total volume of 4.0 µl + 1 µl template genomic DNA with a concentration of 5 ng/µl. The reaction was analysed using an Applied Biosystems 7900 Fast RT‐PCR instrument. Included in the analysis were three negative controls (no template control) and five positive controls (known DNA samples and known SNP assays). The genotyping was validated using a set of five control samples with genotype data available through the Coriell Institute for Medical Research. There was a 100% concordance with the data from Coriell Institute for Medical Research.

We observed an allele frequency distribution of 0.475 Met and 0.525 Val in adolescents, 0.515 Met and 0.485 Val in adults, and 0.491 Met and 0.509 Val in the whole sample, which is comparable to previously reported allele frequency distributions of 0.48 Met and 0.52 Val (Hapmap European sample, http://www.ncbi.nlm.nih.gov/SNP/snp_ref.cgi?rs=4680). The allelic distribution of *COMT* was in Hardy‐Weinberg equilibrium in adolescents (χ^2^(1) = 0.91, *p* = .341), adults (χ^2^(1) = 1.27, *p* = .260) and the whole sample (χ^2^(1) = 2.17, *p* = .140).

### 2.3 Behavioural assessments

Participants were tested on the three WM tasks in this order: (a) social trait‐ranking WM task (Meyer et al., 2012), (b) visuospatial WM grid task (Dumontheil et al., 2011); and (c) backwards digit span task. The social trait‐ranking WM task and visuospatial WM were computerized and developed in MatLab with experimental stimuli designed in Cogent graphic (http://www.vislab.ucl.ac.uk/cogent_graphics.php). Two additional computerized tasks, not described here, were performed by the participants between the first two WM tasks (see Kilford et al., 2015; Magis‐Weinberg et al., 2017 for analyses of these tasks). The testing session ended with the completion of the vocabulary subtest of the WASI (Wechsler, 1999) and collection of the saliva sample, taking approximately 1 hr in total.

#### 2.3.1 Backwards digit span task

The backwards digit span task measures verbal WM for numerical information. Participants were presented with sequences of digits of increasing load (number of digits in the sequence), which they had to repeat in the reverse order. There was a maximum of four trials at loads 3, 4 and 5 and two trials at load 7. Correct reversal of three of four trials was required to start the next load level. The score was the total number of correct reversals, out of a total of 14 trials. One adult participant had a score of 0, which was further than 3 *SD* away from the mean score over the whole sample, and was therefore excluded (final *n* = 332).

#### 2.3.2 Visuospatial WM task

The visuospatial WM task measures spatial WM for visually presented stimuli and was adapted from the Dot Matrix test of the Automated WM Assessment (Alloway, 2007). The task required participants to remember and replicate the order and location of sequences of dots presented one by one in a four by four grid. Each dot was presented for 600 ms, with a 300 ms interval between dots. Each sequence of dots was followed by a short delay (1.5 s), after which participants reproduced the sequence using a computer mouse. Trials varied in load depending on the number of dots in a sequence (between three and eight). There were four trials of each load condition and correct reversal of three trials was required to start the next load level. The score was the total number of correct sequence reproduction, out of a total of 24 trials. Reaction time (RT) was recorded from the beginning of the response phase to the last response and divided by the number of dots in the trial. Data were overwritten and lost for one adolescent participant. There were no outliers on the visuospatial WM score (final *n* = 332), however, three participants (two adolescents, one adult) were slower than 3 *SD* above the mean visuospatial WM RT and were excluded from analyses including this measure (final *n* = 329).

#### 2.3.3 Social trait‐ranking WM task

**Figure 1:**
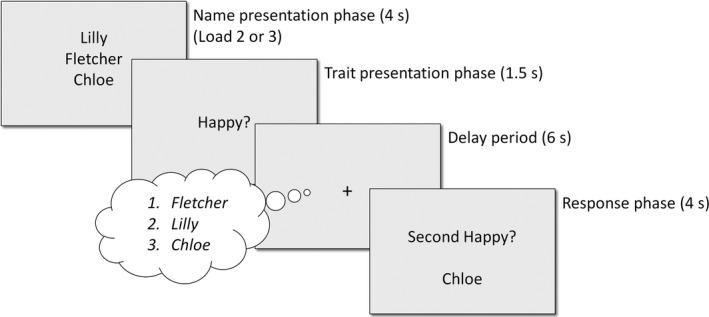
Social trait‐ranking working memory paradigm. Schematic description of the four phases of a load 3 trial, including timings

The social trait‐ranking WM task is a recently developed task that uses social stimuli within a standard WM task paradigm (Meyer et al., 2012). Prior to the study, participants completed a questionnaire in which they named and rated 10 friends on 10 predefined personality traits (e.g., funny, clever, helpful), using a rating scale from 0 to 100. Forty trials were generated by combining the names of friends whose ratings varied by at least five points on a given personality trait. Trials were equally distributed between load 2 (two names) and load 3 (three names). On each trial, participants were first presented with a list of names, followed by a personality trait (e.g., “happy”). During a delay period, participants were asked to order, in a decreasing manner, in their head, the names on the list according to how much the personality trait applied to each of the names (i.e., the happiest friend would be at the top of the list; see Figure 1). We then collected a measure of participants’ WM manipulation by presenting a question such as “Second happy? Chloe”, which required a yes/no response using a right/left index finger key press. Participants were asked to answer as quickly and accurately as possible. Measures of accuracy (consistency between questionnaire and responses in the task) and RT (mean response time to the final question, averaged across loads 2 and 3) were calculated. Data were lost for 15 adolescent participants, not collected for six adolescent participants who did not complete the necessary initial questionnaire (one of whom also did not provide a saliva sample), and incomplete for one adolescent participant because of a computer malfunction (final *n* = 312). In addition, there were no outliers on accuracy, but two adult participants responded on average faster than 3 *SD* below the mean RT and were excluded (final *n* = 310).

### 2.4 Statistical analyses

Statistical analyses were carried out in SPSS version 24. Univariate ANOVAs were performed to investigate the effects of genotype (Met/Met, Val carriers) and age group (children and adolescents, adults) on task performance. Analyses were limited to the three measures which showed associations with *COMT* genotype in the adult sample (Dumontheil et al., 2014) to reduce the number of tests performed. Thus, the dependent variables were backwards digit score, visuospatial WM score and social WM mean RT. Bonferroni correction for three analyses led to a statistical threshold of *p* < .016. Gender was included as a between subject factor in all analyses. In addition, analyses were repeated by including ethnicity (Caucasian/non‐Caucasian) as a covariate. All genetic effects remained significant (see Table 2), therefore, we report in the text and plot in relevant figures the estimated standardized means and standard errors obtained from the original ANOVAs (*M* ± *SE*).

**Table 2:** Results of univariate ANOVAs including age group, genotype and gender as independent variables for the key measure of each of the three tasks

	Backwards digit span score (*n* = 332)	Visuospatial WM score (*n* = 332)	Social WM mean RT (*n* = 310)
Homogeneity of variance (Levene's test)	n.s., *p* = .060	n.s., *p* = .616	n.s., *p* = .851
Age group	*F*(1,324) = 67.00, *p* < .001, η*_p_* ^2^ = 0.171	*F*(1,324) = 19.16, *p* < .001, η*_p_* ^2^ = 0.056	*F*(1,302) = 28.67, *p* < .001, η*_p_* ^2^ = 0.087
Genotype	n.s., *p* = .242	n.s., *p* = .223	n.s., *p* = .230
Gender	*F*(1,324) = 4.24, *p* = .040, η*_p_* ^2^ = 0.013^a^	n.s., *p* = .804	n.s., *p* = .517
Age group × genotype	n.s., *p* = .091^b^	*F*(1,324) = 11.09, *p* = .001, η*_p_* ^2^ = 0.033^b^	*F*(1,302) = 7.51, *p* = .007, η*_p_* ^2^ = 0.024^b^
Age group × gender	n.s., *p* = .411	n.s., *p* = .571	n.s., *p* = .812
Genotype × gender	n.s., *p* = .430	n.s., *p* = .562	n.s., *p* = .980
Age group × genotype × gender	n.s., *p* = .808	n.s., *p* = .927	n.s., *p* = .134

a The main effect of gender does not survive Bonferroni correction for three analyses (*p* < .016).

b When ethnicity (Caucasian/not Caucasian) was entered as a covariate the results were as follow: backwards digit span score: n.s., *p* = .083; visuospatial WM score: *F*(1,319) = 9.87, *p* = .002, η*_p_* ^2^ = 0.030; social WM mean RT: *F*(1,297) = 7.03, *p* = .008, η*_p_* ^2^ = 0.023.

Two‐way interactions between age group and genotype, the interaction of interest, were followed up using simple effects analysis (Howell, 1997), with the prediction of an absent or inverted direction of the genotype effect in the developmental sample compared to the adult sample. We also split the sample by genotype to explore whether there were differences between age groups for each genotype. Finally, in order to investigate whether genetic effects on the social WM mean RT could be accounted by genetic effects on standard WM tasks, we repeated the social WM mean RT analyses and entered backwards digit span score, visuospatial WM score and visuospatial WM RT as covariates (Dumontheil et al., 2014).

## 3 RESULTS

### 3.1 Backwards digit span task

**Figure 2:**
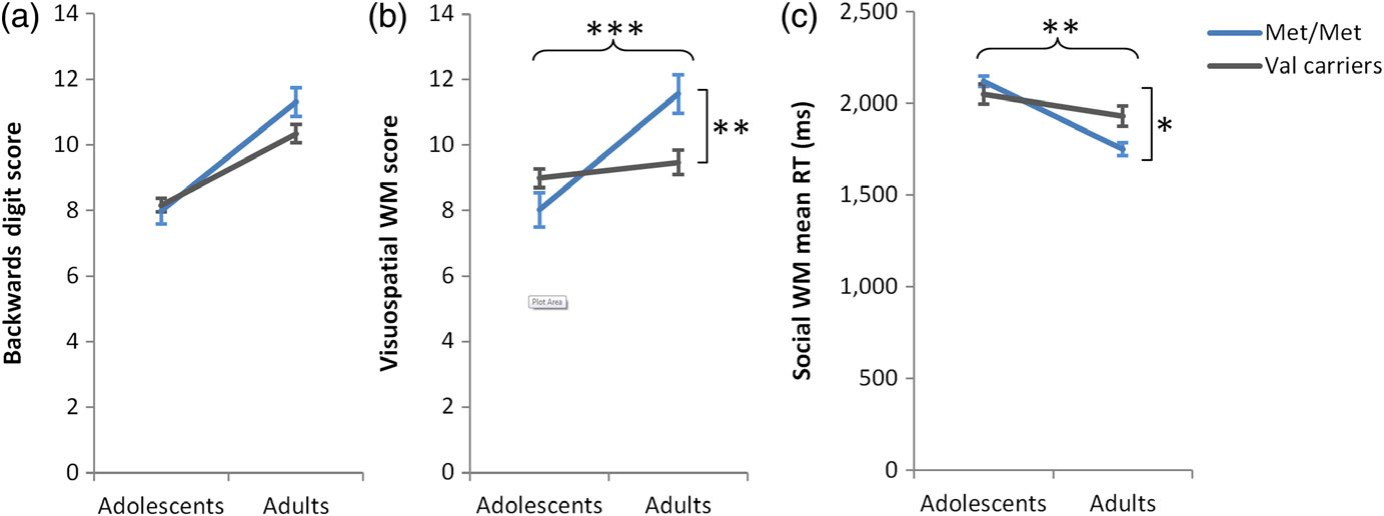
Performance in the (a) backwards digit span task, (b) visuospatial working memory (WM) task and (c) social trait‐ranking WM task as a function of age group and *COMT* genotype. Shown here are estimated means and *SE* from the univariate ANOVAs, which included gender as a factor. The interaction between age group and *COMT* genotype was significant for the visuospatial (b) and social (c) WM tasks. Significant interactions were followed up by analysing the simple main effects. On both the visuospatial (b) and social (c) WM tasks, adult Met/Met individuals significantly outperformed adult Val carriers, whereas this effect was not observed in adolescents. *p < .05, **p < .01, ***p ≤ .001

A univariate ANOVA with backwards digit span score as the dependent variable and age group, genotype and gender as independent variables revealed main effects of age group and gender (Table 2) with better performance in adults (10.82 ± 0.26; estimated marginal means ± *SE*) than adolescents (8.06 ± 0.22) and in females (9.79 ± 0.24) than males (9.09 ± 0.24). There was no significant main effect of genotype and the interaction between age group and genotype was not significant (Table 2, Figure 2a).

### 3.2 Visuospatial WM task

The same analysis was performed for the visuospatial WM task data. Results showed again a significant main effect of age group, with better performance in adults (10.52 ± 0.35) than adolescents (8.51 ± 0.30) but no main effect of gender. The predicted interaction between age group and genotype was significant (Table 2). Simple effects analysis, indicated that the simple main effect of genotype was significant in adults (*F*(1,324) = 8.95, *p* = .003, η*_p_* ^2^ = 0.027, see Dumontheil et al., 2014), with better performance in Met/Met individuals than Val carriers, but not in adolescents (*F*(1,324) = 2.64, *p* = .106) where the direction of effects was in the opposite direction (Figure 2b). Analysis of the simple main effect of Age group indicated that Met/Met adults had higher visuospatial WM scores than Met/Met adolescents (*F*(1,324) = 20.14, *p* < .001, η*_p_* ^2^ = 0.059), while Val carriers adults and Val carriers adolescents did not differ (*F*(1,324) = 1.04, *p* = 0.308).

### 3.3 Social trait‐ranking WM task

Analyses of the mean RT on the social trait‐ranking WM task showed very similar results to the analysis of the visuospatial WM task. There was a main effect of age group (Table 2), with faster RTs in adults (1,840 ms ± 34) than adolescents (2,085 ms ± 31), and a significant interaction between age group and genotype (Table 2). Similar to visuospatial WM, analysis of simple effects indicated that the effect of genotype was significant in adults (*F*(1,302) = 7.12, *p* = .008, *η*_p_^2^ = 0.023, see Dumontheil et al., 2014), with faster RTs in Met/Met individuals than Val carriers, but not in adolescents (*F*(1,302) = 1.30, *p* = .255), where the pattern was in the opposite direction (Figure 2c). Simple effects analyses also indicated that the difference in social WM RT between Met/Met adults and Met/Met adolescents was greater (*F*(1,302) = 22.10, *p* < .001, *η*_p_^2^ = 0.068), than between adult and adolescent Val carriers (*F*(1,302) = 6.60, *p* = .011, *η*_p_^2^ = 0.021; Figure 2c), a pattern similar to that observed in the visuospatial WM task. The interaction between genotype and age group remained significant when covarying for backwards digit score (*p* = .010), visuospatial WM score (*p* = .028; note this interaction does not survive Bonferroni correction for three analyses at *p* < .016) and visuospatial WM mean RT (*p* = .006), as did the main effect of age group (*p*'s < .001).

## 4 DISCUSSION

This is the first study using a common genetic polymorphism affecting the function of the COMT enzyme to probe the development the dopamine neurotransmitter system, through its involvement in social and non‐social WM. In a previous study, we demonstrated that variation at *COMT* was associated with performance on verbal and visuospatial WM tasks in adults (Dumontheil et al., 2014), findings consistent with the suggestion that Met/Met individuals have levels of dopamine in the PFC suitable for optimal WM performance (Meyer‐Lindenberg & Weinberger, 2006). Using a novel social WM paradigm, which requires participants to maintain and manipulate information about the traits of their family and friends over a delay, in our previous study we further demonstrated that social WM performance was also associated with variation at *COMT*, and that this association was not fully accounted for by individual differences in verbal or visuospatial WM (Dumontheil et al., 2014). Here, data collected in children and adolescents were compared with the data from this adult sample to investigate whether the genetic associations were observed across ages. Our results replicate and extend previous work showing that the pattern of better WM performance in Met/Met individuals observed in adulthood emerges during development, which is consistent with a decrease in the levels of prefrontal dopamine during adolescence (Dumontheil et al., 2011; Jucaite et al., 2010; Wahlstrom et al., 2007).

Our first aim was to replicate the finding that the association between *COMT* genotype and visuospatial WM performance is not stable throughout childhood, adolescence and adulthood (Dumontheil et al., 2011; Wahlstrom et al., 2007; Wahlstrom, Collins, et al., Wahlstrom, Collins, et al., 2010; Wahlstrom, White, et al., 2010), reflecting underlying changes in the dopamine neurotransmitter system (Jucaite et al., 2010; Wahlstrom, White, et al., 2010). Indeed, we observed an interaction between *COMT* genotype and age group on the visuospatial WM task score, whereby while the Val allele was associated with poorer visuospatial WM in adulthood, this was not the case in childhood and adolescence. While performance of Val carriers did not differ between age groups, visuospatial WM performance was better in Met/Met adults than Met/Met children and adolescents. These results replicate those observed in a previous study using the same task in a longitudinal and cross‐sectional sample of participants aged 6–25 years old (Dumontheil et al., 2011), where steeper improvements in performance with age were observed in Met/Met individuals than Val carriers, leading to the emergence of the adult pattern of better visuospatial WM performance in Met/Met individuals. It is also consistent with the neuroimaging results from that study, which again showed an interaction between genotype and age, and a gradual emergence of the adult pattern of *COMT* genotype differences in brain activation during executive functions task (e.g. de Frias et al., 2010; Dickinson & Elvevåg, 2009; Mier et al., 2010; Tunbridge et al., 2006; Witte & Flöel, 2012, but see null results of meta‐analysis by Nickl‐Jockschat et al., 2015). The pattern of findings observed in the current study, consistent with previous findings (Dumontheil et al., 2011; Wahlstrom et al., 2007), is that children and adolescents who are Val carriers do not show deficits in visuospatial WM, in line with the improvement in performance observed in Val/Val adults administered amphetamine (Mattay et al., 2003). These results are therefore consistent with a decrease in prefrontal basal dopamine levels during adolescence (Dumontheil et al., 2014; Wahlstrom et al., 2007; Wahlstrom, Collins, et al., Wahlstrom, Collins, et al., 2010; Wahlstrom, White, et al., 2010).

The second aim of the present study was to assess whether a similar developmental difference in *COMT* genotype effect could be observed for a verbal WM task. Although the pattern was similar overall, the genotype by age group interaction was not significant for the backwards digit span task. This may reflect the fact that the verbal and visuospatial WM tasks rely on partially distinct aspects of the PFC, which may differ in their dopaminergic innervation. A meta‐analysis of neuroimaging studies of WM has shown that the caudal superior frontal sulcus appeared specifically sensitive to spatial content, while a left mid‐lateral inferior frontal gyrus region was more sensitive to non‐spatial, in particular verbal, content (Nee et al., 2013). Alternatively, as both human pharmacological studies reviewed above used verbal n‐back WM tasks to demonstrate differential effects of tolcapone or amphetamine as a function of *COMT* genotype (Giakoumaki et al., 2008; Mattay et al., 2003), it is possible that *updating* verbal information in WM, which is necessary in n‐back tasks, may be more dependent on the dopaminergic system than the maintenance and manipulation (with no updating) of verbal information, which is required in the backwards digit span task.

Finally, the third aim was to assess whether there was also an interaction between age and *COMT* genotype on the performance of a social WM task, and the extent to which associations between *COMT* genotype and social WM performance could be accounted for by *COMT*’s association with non‐social WM skills, as opposed to skills specific to the processing of social information. The results show that there was indeed an interaction between *COMT* genotype and age group in the social trait‐ranking WM task, with the adult pattern of faster RT in Met/Met individuals (Dumontheil et al., 2014) emerging with age. As in the visuospatial WM task, this result reflected steeper improvements over development in Met/Met individuals than Val carriers, which may be limited by their basal dopamine levels. Importantly, the interaction between genotype and age group in the social WM task was not fully mediated by individual differences in verbal or visuospatial WM, which is similar to what was observed in the adult sample (Dumontheil et al., 2014).

There were some limitations to this study. First, the adolescent sample included six sibling pairs, which means that the corresponding genetic data cannot be considered as independent. However, these few cases are unlikely to have impacted the results. Second, due to the time constraints of school‐based testing participants only completed the vocabulary subtest of the WASI, which provided an estimate of verbal IQ but not total IQ. We were therefore not able to control for possible differences between age or genotype groups in non‐verbal IQ, which is strongly related, although not identical, to WM (Conway, Cane & Engle, 2003). We made the decision not to analyse social WM accuracy on the basis that this measure did not show an association with *COMT* genotype in the adult sample (Dumontheil et al., 2014). The fact that the main verbal and visuospatial WM measures were mean accuracy scores while the social WM measure was a mean RT may be a limitation to this follow‐up analysis. However, we observed the same effect when mean RT on the visuospatial WM was included as a covariate in place of visuospatial WM accuracy. Furthermore, partial correlation analyses between WM measures (Supplementary Materials, Supplementary Analysis 5) indicated that social WM RT was significantly negatively correlated with accuracy scores on both the standard WM tasks.

The social WM trait‐ranking task has been shown to recruit regions of the social brain, in particular the medial PFC, precuneus and temporo‐parietal junction (Meyer et al., 2012, 2015), in addition to the typical lateral fronto‐parietal regions typically observed in standard verbal or visuospatial WM tasks (Nee et al., 2013; Owen et al., 2005; Rottschy et al., 2012; Van Overwalle, 2009). Our results therefore suggest that similar changes in basal dopamine levels are occurring in the social WM specific brain regions, in particular the medial PFC, as those occurring in the lateral PFC regions supporting non‐social WM. The present finding of parallel developmental changes in WM and social cognition fits with the observation of the prolonged development of cognitive control and social cognition during adolescence (Burnett et al., 2011; Dumontheil, 2016; Luna et al., 2010). More specifically, our results are in line with evidence suggesting that the social and non‐social higher cognitive brain systems tend to be recruited in parallel rather than showing interactions during development (e.g. Dumontheil et al., 2012; Magis‐Weinberg et al., 2017).

There was a main effect of gender on performance in the verbal WM task (although it did not survive Bonferroni correction), however no other main effect and no interaction with age group or genotype was observed. There is some evidence that rs4680 associations with behaviour, brain structure and the incidence of psychiatric disorders may interact with gender (Gogos et al., 1998; Harrison & Tunbridge, 2008). Oestrogens, which are thought to downregulate COMT activity, may be behind these gender differences (Gogos et al., 1998; Harrison & Tunbridge, 2008). Studies with larger numbers and puberty measures may be needed to detect gender differences and to further our understanding of the possible role of gender differences in the development of the dopamine neurotransmitter system.

Future studies could also demonstrate a greater specificity of the association between *COMT* genotype and social WM by including a non‐social WM task more closely matched to the social trait‐ranking task (Meyer et al., 2015). By including an updating verbal WM task, such as the n‐back task, they could also assess whether updating verbal information in WM is more dependent on the dopamine neurotransmitter system than the maintenance and manipulation of verbal information measured in the backwards digit span task. The results of the present study suggest that the range of WM tasks used in the literature, as well as differences in the age of participants, may account for some of the inconsistencies in findings previously obtained (Barnett et al., 2008; Dickinson & Elvevåg, 2009; Witte & Flöel, 2012).

In sum, the present study shows that associations between *COMT* genotype and task performance change during development in a range of WM measures. Met/Met individuals show steeper improvements in performance between age groups than Val carriers, suggesting that the progress of Val carriers may be limited by their basal dopamine levels. In line with this, previous neuroimaging data suggest that top‐down excitation from frontal to parietal cortex may be increased during adolescence in Val/Val individuals to compensate for suboptimal levels of dopamine and parietal functioning (Dumontheil et al., 2011; Edin et al., 2009), leading to the increased PFC activation observed, although inconsistently, in executive function tasks in Val carriers compared to Met/Met individuals in adulthood (Dickinson & Elvevåg, 2009; de Frias et al., 2010; Mier et al., 2010; Nickl‐Jockschat et al., 2015; Tunbridge et al., 2006; Witte & Flöel, 2012). Many psychiatric conditions first appear during adolescence (Paus, Keshavan, & Giedd, 2008) and have been associated with atypical functioning of the dopamine neurotransmitter system and genotypic variation in dopamine‐related genes (Meyer‐Lindenberg & Weinberger, 2006). It is therefore important to better understand how genetic variation affects the development of brain and cognition during adolescence, as this could in turn inform our understanding of adolescent behaviour as well as the emergence of psychiatric disorders. Indeed, the findings presented here show that development should be considered when trying to understand the impact of genetic polymorphisms on the mature higher cognition of healthy adult or psychiatric populations.

## CONFLICT OF INTEREST

The authors declare no conflict of interest.

## Data Availability

The data that support the findings of this study are available from the corresponding author, Dr. Iroise Dumontheil (i.dumontheil@bbk.ac.uk) upon reasonable request.
