# Supplementary material for: Development of dopaminergic genetic associations with visuospatial, verbal and social working memory
Source: Dev Sci. 2019 Aug 6;23(2):e12889. doi: 10.1111/desc.12889 (PMC7064996; doi:10.1111/desc.12889)
Supplement: Supplementary file 1 [file DESC-23-e12889-s001.docx]

**Supplementary Materials for *Development of dopaminergic genetic associations with visuospatial, verbal and social working memory***

Iroise Dumontheil, Emma J Kilford, Sarah-Jayne Blakemore

**Supplementary Analysis 1: Age matching of adult *COMT* genetic groups**

One-way ANOVAs indicated that the genotype groups (Met/Met vs. Val carriers) were matched in the adolescent group (*F*(1,200) = 0.203, *p* = .653), while Val carriers were significantly older than Met/Met individuals in the adult group (*F*(1,129) = 5.120, *p* = .025; see **Methods** and **Table 1** in the main text). To emphasise the fact that our findings were not influenced by age differences in the adult genotype groups an additional analysis was performed, in which adult *COMT* groups were matched for age.

The age difference between the adult genotype groups was driven by the fact that only two Met/Met participants were older than 29 years old (33.9 and 35.9 years old) while 23 Val carriers were 29 years or older. When all participants aged 29 years old or older were excluded from the sample, the age difference between Met/Met and Val carriers in the adult sample was no longer significant (*F*(1,105) = 0.397, *p* = .530). When the main analyses were repeated on this age-matched sample, the results remained very similar (there were no qualitative changes, just very small changes in *p*-values and effect sizes), in particular for the age group x *COMT* interaction, as shown in **Table S1.**

**Table S1:** Comparison of interactions between age group and *COMT* genotype between the main text analyses and analyses in which participants aged 29 years or older were excluded to match the ages of the adult *COMT* genotype groups.

| ***Age group x COMT genotype*** | **Original analyses** | **Analyses excluding participants 29y or older** |
| --- | --- | --- |
| Backwards digit score | n.s., *p* = .091 | n.s. *p* = .089 |
| Visuospatial WM score | *p* = .001, η*_p_*^2^ = .033 | *p* = .004, η*_p_*^2^ = .028 |
| Social WM mean RT | *p* = .007, η*_p_*^2^ = .024 | *p* = .006, η*_p_*^2^ = .026 |

**Supplementary Analysis 2: Continuous age effects across the sample**

Additional analyses were performed to investigate interactions between *COMT* genotype and age considered as a continuous measure. Previous research suggests that improvements in WM performance over the lifespan are not linear, but instead increase during adolescence before starting to decline from the 20s (Cansino et al., 2013; Hale et al., 2011; a quadratic effect of age) or may plateau in the 20s and 30s (Alloway & Alloway, 2013; Dumontheil et al., 2011; an inverse model of age). Thus, in a first step, we first used standardised model comparison procedures to examine whether the relationship between age and each of the WM measures was best explained by a linear, quadratic or inverse model of age. Goodness of fit was compared using the Akaike information criterion (AIC; Akaike, 1974) and Bayesian information criterion (BIC; Schwarz, 1978), standardised criteria enabling the comparison of model fit, in which lower values indicate superior model fit.

AIC and BIC results indicated that for all three WM measures the inverse and quadratic models were of a better fit than the linear model.^[[1]](#footnote-1)^ For the backwards digit and visuospatial WM scores the inverse and quadratic models were of similarly good fit, while the quadratic model was a better fit for the social WM mean RT measure (see **Table S2** for AIC and BIC values). To facilitate comparison between the three tasks, two series of multiple regression analyses were therefore performed using both inverse and quadratic functions of age (standardised by *z* scores: age_Z_) for all three tasks.

In a second step, models including effects of age, sex and *COMT* genotype (Met/Met, Val carriers), and their interactions were run to assess whether the main effect of sex and interactions with sex were significant. None of the interaction terms was significant, but sex was a significant predictor of backwards digit score. Sex was therefore entered as a regressor in the analyses of this measure, but not in analyses of visuospatial or social WM.

Thus, the first series of final models (A1:A3) included the inverse of age (age^-1^_Z_), *COMT* genotype and the interaction between the inverse of age and genotype as regressors (and sex for backwards digit score). All predictors were entered in a single step. Both age^-1^_Z_ and the interaction between age^-1^_Z_ and genotype were found to be significant for all three WM measures (**Table S1,** and **Figure S1A, S1B, S1C, S1E**).

In the second series of models (B1:B3) both linear (age_Z_) and quadratic (age_Z_^2^) regressors were included, along with *COMT* genotype and the interactions between genotype and the two age regressors. Both age_Z_ and age_Z_^2^ were significant predictors of all three WM measures, and the interaction between age_Z_ and genotype was significant for the visuospatial WM score, and at trend (*p* = .053) for the social WM mean RT (**Table S1**, **Figures S1D, S1F**). However the interaction between age_Z_^2^ and genotype was not significant, therefore although all predictors were entered in a single step, for simplicity we re-ran the models excluding this term.

**Table S2:** Results of multiple regression analyses of performance on the three working memory tasks as a function of age and *COMT* Val dominant genotype. Models A1, A2, A3 use an inverse function of age, while models B1, B2, B3 use a quadratic function of age. All predictors were entered in a single step in the final models.

|  | **Backwards digit score** | | **Visuospatial WM score** | | **Social WM mean RT** | |
| --- | --- | --- | --- | --- | --- | --- |
|  | **Model A1** | | **Model A2** | | **Model A3** | |
| Inverse of age models | *F*(4,327) = 27.82,  *p* < .001, *R*^2^ = 25.4%  AIC = 626, BIC = 638^b^ | | *F*(3,328) = 11.13,  *p* < .001, *R*^2^ = 9.2%  AIC = 842, BIC = 853^c^ | | *F*(3,306) = 13.01,  *p* < .001, *R*^2^ = 11.3%  AIC = 3620, BIC = 3631^d^ | |
|  | β | *p*-value | β | *p*-value | β | *p*-value |
| Sex | -.156 | .001 |  |  |  |  |
| Age^-1^_Z_ | -.665 | <.001 | -.534 | <.001 | .553 | <.001 |
| Genotype^a^ | -.067 | .165 | -.047 | .376 | .046 | .397 |
| Age^-1^_Z_ x genotype^a^ | .209 | .028 | .331 | .002 | -.297 | .006 |
|  | **Model B1** | | **Model B2** | | **Model B3** | |
| Quadratic age models | *F*(5,326) = 22.12, *p* < .001, *R*^2^ = 25.3%  AIC = 624, BIC = 639^b^ | | *F*(4,327) = 9.23, *p* < .001, *R*^2^ = 10.1%  AIC = 839, BIC = 854^c^ | | *F*(4,305) = 13.83, *p* < .001, *R*^2^ = 15.4%  AIC = 3603, BIC = 3618^d^ | |
|  | β | *p*-value | β | *p*-value | β | *p*-value |
| Sex | -.141 | .004 |  |  |  |  |
| Age_Z_ | .745 | <.001 | .611 | <.001 | -.606 | <.001 |
| Age_Z_^2^ | -.272 | <.001 | -.211 | .002 | .347 | <.001 |
| Genotype^a^ | -.057 | .238 | -.039 | .463 | .026 | .620 |
| Age_Z_ x genotype^a^ | -.170 | .099 | -.330 | .004 | .220 | .053 |

^a^ *COMT* genotype: 0: Met/Met; 1: Val carriers

^b^ Linear age model: AIC = 644, BIC = 656

^c^ Linear age model: AIC = 851, BIC = 862

^d^ Linear age model: AIC = 3633, BIC = 3645


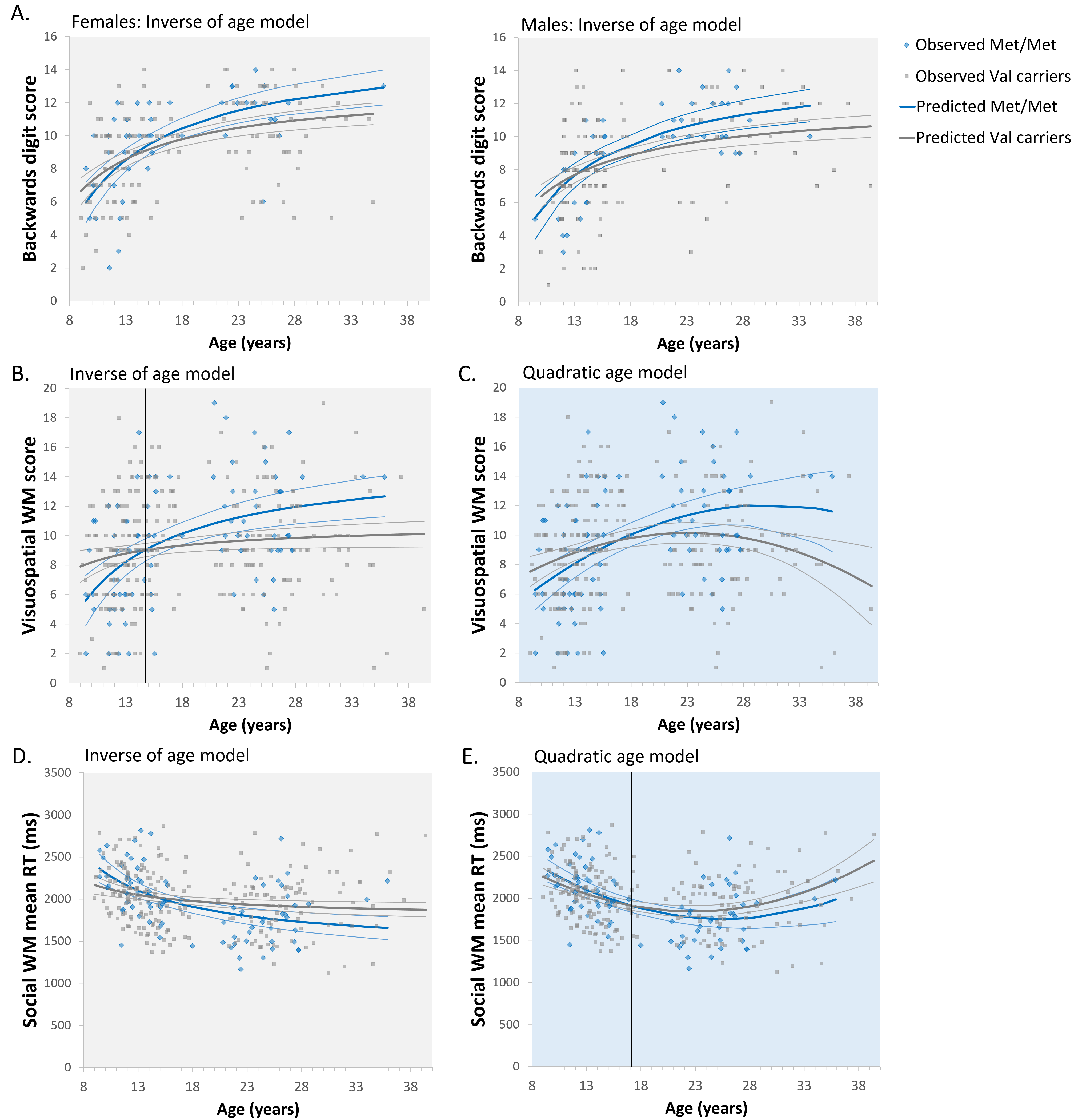


**Figure S1:** Scatterplots of performance on the backwards digit (**A**), visuospatial working memory (WM) (**B,C**) and social trait ranking WM tasks (**D,E**) as a function of age and *COMT* genotype. Lines show unstandardised predicted values with confidence intervals for WM performance calculated from the regression models summarised in **Table S1** for Met/Met (blue) and Val carriers (grey). When age was modelled using an inverse function (grey background, **Table S1,** models A1:A3), there was a significant effect of age, and a significant interaction between *COMT* and age for all three measures: backwards digit score (**A,** split by sex); visuospatial WM score (**B**); social WM mean RT (**D**). Only backwards digit score is plotted separately for each sex, because sex was included as an additional regressor in this model. When age was modelled using a quadratic function (blue background, **Table S1,** models B1:B3), there was a significant quadratic effect of age for all three tasks, and also a significant interaction between the linear age term and *COMT* genotype for the visuospatial WM measure (**C**), while this interaction was at trend for the social WM measure (**E**). The approximate cross-over points between the predicted lines for Met/Met and Val carriers genotypes are indicated by a vertical line on each graph.

To summarise these analyses, performance on all three WM tasks showed non-linear changes with age, with increases in performance during adolescence which plateaued in the twenties or worsened in the thirties. Consistent with the age group analyses reported in the main paper, there were significant interactions between age and *COMT* genotype on the visuospatial WM and social WM measures using inverse of age models. This pattern was also observed in the backwards digit span task measure, where the *COMT* genotype x age group interaction was only at trend in the main paper. The approximate cross-over point between the fit lines for each genotype occurred earlier (around 13 years old; **Figure S1A, S1B**) for the backwards digit score than for the visuospatial WM score and social WM mean RT, which were similar (around 15 years old; **Figures S1C, S1E**). When using quadratic age models, there was an interaction between the linear age term and *COMT* genotype which was significant in the visuospatial WM task only, with only trends in the other two tasks. The cross-over for the visuospatial WM and social WM tasks occurred around 17 years old (**Figures S1D, S1F**). Overall these results are consistent in showing that the effect of *COMT* genotype on WM performance changes over the course of development, with the adult pattern of Met/Met advantage only emerging in late adolescence.

**Supplementary Analysis 3: Continuous age effect in the adolescent sample**

As curve-fitting approaches may not fully capture developmental trajectories, and as there was a gap in the age distribution between our adolescent and adult samples, a second series of analyses focused on the adolescent sample only. Again, multiple regressions were performed to first assess whether sex and interactions with sex were significant predictors. If there were not, they were not included in the final model. Predictors in the final model were entered in a single step. A linear model of age effects was used.

None of the interaction terms where significant, but sex was a significant predictor of backwards digit score and social WM mean RT. Sex was therefore entered in the analyses of these measures. Age was a significant predictor for all three dependent variables (backwards digit score: β = .543; visuospatial WM score: β = .395; social WM mean RT: β = -.479; all *p*’s < .005), with improving performance with age. Sex was a significant predictor for the backwards digit score (β = -.232; *p* = .001) and social WM mean RT (β =.205; *p* = .005), with better performance by female than male children. *COMT* Val dominant genotype did not explain additional variance in WM tasks performance (backwards digit score: *p* = .215; visuospatial WM score: *p* = .151; social WM mean RT: *p* = .140), nor did the interaction term between *COMT* genotype and age (backwards digit score: *p* = .159; visuospatial WM score: *p* = .592; social WM mean RT: *p* = .439).

**Supplementary Analysis 4: Co-dominance model of *COMT* rs4680 alleles**

We made the *a priori* decision to model effects of *COMT* genotype using the Val allele dominant genotype grouping (0: Met/Met, 1: Val carriers) that was found to show association with performance in our analyses of the adult data (Dumontheil et al., 2014), and has been shown in previous studies to be the most effective model for explaining the influence of *COMT* variance on behaviour (Barnett et al., 2008; Dumontheil et al., 2011). However, to facilitate comparison with the results of other studies, we also present the results of a *post hoc* exploratory analysis in which all three *COMT* genotypes (0: Met/Met; 1: Val/Met; 2: Val/Val) were compared, allowing for possible co-dominance of the two alleles. As in the main text, univariate ANOVA’s were performed including sex, genotype and age group as between subjects factors. Two-way interactions between age group and genotype, the interaction of interest, were followed up using simple effects analysis (Howell, 1997). Effects of genotype were followed up using Bonferroni-corrected pairwise comparisons.

The pattern of results for all effects was found to be consistent with those presented in the main analysis using a Val dominant model (see **Table S3, Figure S2**). As in the main text, there was no significant interaction between age group and genotype on backwards digit score, however the interaction was significant for both visuospatial WM score and social WM mean RT. For visuospatial WM score, simple main effects analysis indicated a significant effect of *COMT* genotype in adults (*F*(2,320) = 5.08, *p* = .007, η*_p_*^2^ = .031), but not in adolescents (*F*(2,320) = 1.44, *p* = .239; **Figure S2B**). Bonferroni-corrected pairwise comparisons indicated that Met/Met adults performed better than

Val/Val adults (*p* = .002) and marginally better than Val/Met adults (*p* = .069). Val/Met and Val/Val adults did not significantly differ in performance (*p* = .702). Analysis of the simple main effect of age group indicated that Met/Met adults had higher visuospatial WM scores than Met/Met adolescents (*F*(1,320) = 20.24, *p* < .001, η*_p_*^2^ = .059), whereas Val/Met and Val/Val adults did not significantly differ from their adolescent counterparts (Val/Met: *F*(1,320) = 2.18, *p* = .141; Val/Val: *F*(1,320) = 0.06, *p* = .803).

**Table S3:** Results of univariate ANOVAs including age group, *COMT* genotype (Met/Met; Val/Met; Val/Val) and sex as independent variables for the key measure of each of the three tasks.

|  | | **Backwards digit score (*n* = 332)** | **Visuospatial WM score (*n* = 332)** | **Social WM mean RT  (*n* = 310)** |
| --- | --- | --- | --- | --- |
| **Homogeneity of variance (Levene’s test)** | *F*(11,320) = 1.82,  *p* = .050 | | *F*(11,320) = 0.62,  *p* = .811 | *F*(11,298) = 0.46,  *p* = .926 |
| **Age group** | *F*(1,320) = 68.97, *p* < .001, η*_p_*^2^ = .177 | | *F*(1,320) = 11.37,  *p* = .001, η*_p_*^2^ = .034 | *F*(1,298) = 26.17,  *p* < .001, η*_p_*^2^ = .081 |
| **Genotype** | n.s., *p* = .422 ^a^ | | n.s., *p* = .378 ^b^ | n.s., *p* = .325 ^c^ |
| **Sex** | *F*(1,320) = 3.93,  *p* = .048, η*_p_*^2^ = .012 ^d^ | | n.s., *p* = .818 | n.s., *p* = .499 |
| **Age group x genotype** | n.s., *p* = .243 ^e^ | | *F*(2,320) = 6.17,  *p* = .002, η*_p_*^2^ = .037 ^e^ | *F*(2,298) = 4.74,  *p* = .009, η*_p_*^2^ = .031^e^ |
| **Age group x sex** | n.s., *p* = .378 | | n.s., *p* = .847 | n.s., *p* = .414 |
| **Genotype x sex** | n.s., *p* = .741 | | n.s., *p* = .505 | n.s., *p* = .915 |
| **Age group x genotype x sex** | n.s., *p* = .965 | | n.s., *p* = .120 | n.s., *p* = .328 |

^a^ n = 332: 49 Met/Met, 94 Met/Val, 59 Val/Val adolescents; 38 Met/Met, 59 Met/Val, 33 Val/Val adults

^b^ n = 332: 49 Met/Met, 94 Met/Val, 58 Val/Val adolescents; 38 Met/Met, 59 Met/Val, 34 Val/Val adults

^c^ n = 310: 45 Met/Met, 83 Met/Val, 53 Val/Val adolescents; 37 Met/Met, 58 Met/Val, 34 Val/Val adults

^d^ The main effect of Sex does not survive Bonferroni correction for three analyses (*p* < .016)

^e^ When ethnicity (Caucasian/not Caucasian) was entered as a covariate the results were as follows: backwards digit span score: n.s., *p* = .221; visuospatial WM score: *F*(2,315) = 5.39, *p* = .005, η*_p_*^2^ = .033; social WM mean RT: *F*(2,293) = 4.33, *p* = .014, η*_p_*^2^ = .029.

For social WM mean RT, simple main effects analysis again indicated a significant effect of *COMT* genotype in adults (*F*(2,298) = 4.67, *p* = .010, η*_p_*^2^ = .030), but not in adolescents (*F*(2,298) = 0.74, *p* = .479; **Figure S2C**). Bonferroni-corrected pairwise comparisons indicated that Met/Met adults performed better than Val/Met adults (*p* = .009) and did not significantly differ from Val/Val adults (*p* = .537). Again, Val/Met and Val/Val adults did not significantly differ in their performance (*p* = .405). Analysis of the simple main effect of Age group indicated that Met/Met adults had faster mean social RTs than Met/Met adolescents (*F*(1,298) = 21.99, *p* < .001, η*_p_*^2^ = .069), as did Val/Val adults relative to Val/Val adolescents (*F*(1,298) = 6.86, *p* = .009, η*_p_*^2^ = .023), whereas Val/Met adults did not significantly differ from Val/Met adolescents (*F*(1,298) = 1.33, *p* = .250).


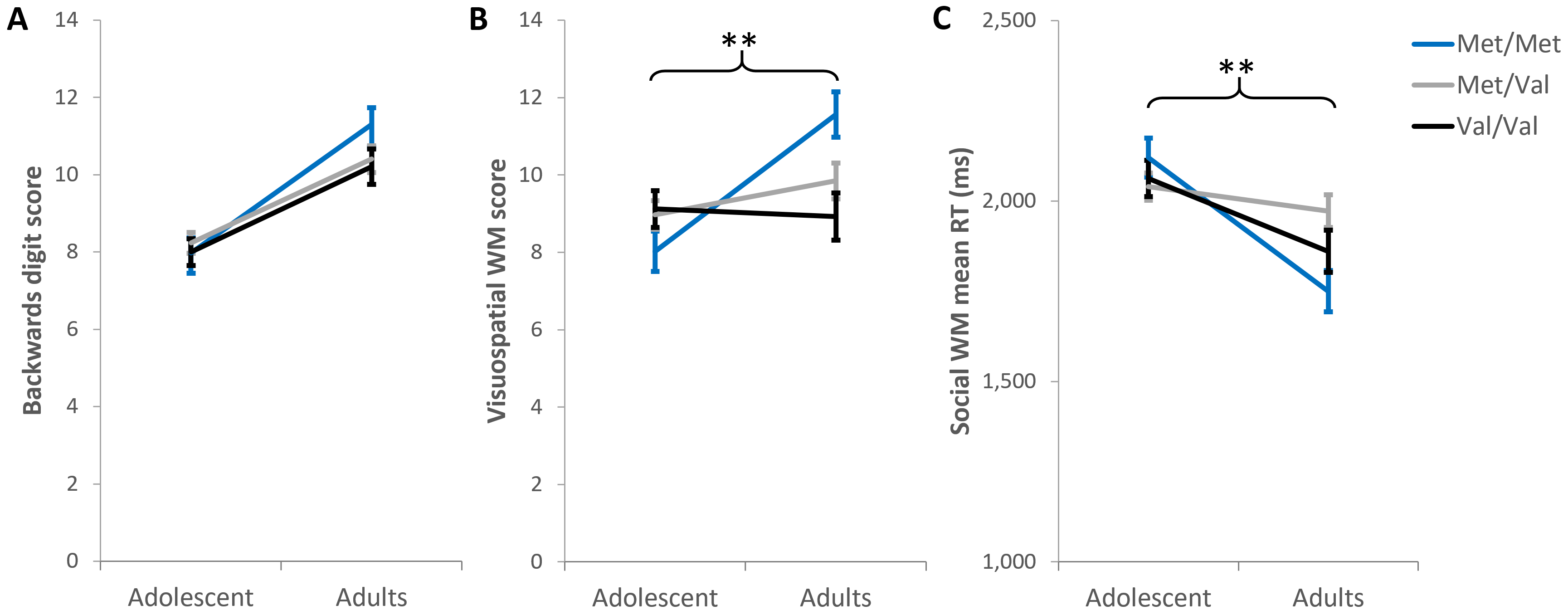


**Figure S2:** Performance in the (**A**) backwards digit span task, (**B**) visuospatial WM task and (**C**) social trait-ranking WM task as a function of age group and the three *COMT* genotypes. Shown here are estimated means and SE from the univariate ANOVAs, which included sex as a factor. The interaction between age group and *COMT* genotype was significant for the visuospatial **(B)** and social **(C)** WM tasks. Significant interactions were followed up by analysing the simple main effects. On both the visuospatial **(B)** and social **(C)** WM tasks, adult Met/Met individuals significantly outperformed adult Val carriers, whereas this effect was not observed in adolescents. * *p* < .05, ** *p* < .01, *** *p* ≤ .001.

Overall these results are consistent with the main analyses, and do not suggest co-dominance of the Val and Met allele for visuospatial WM or social WM tasks performance.

**Supplementary Analysis 5: Correlations**

Analyses in the main text were limited to the three measures which previously showed associations with *COMT* genotype in adults (Dumontheil et al., 2014) to reduce the number of tests performed. Therefore, we did not investigate age x genotype interactions for either VSWM RT or social WM accuracy. Supplementary analyses were performed to assess associations between accuracy and RT measures in the three working memory tasks, and in particular test whether social WM mean RT, although a RT rather than accuracy measure, was associated with scores on the standard WM tasks. Partial correlations were run between the measures, controlling for age and gender.

As can be seen in **Table S4** total accuracy scores on the two standard WM paradigms were moderately correlated (controlling for age and sex), indicating that participants who performed well on one standard WM task also performed well on the other (**Table S4**). VSWM RT was significantly positively correlated with VSWM score, indicating that greater accuracy was associated with slower task performance. VSWM RT was not correlated with any other WM measures. In contrast, social WM RT was significantly negatively correlated with accuracy scores on both the standard WM tasks (**Table S4**), suggesting that this measure was reflecting the cognitive processes facilitating social WM in this task, with participants showing greater accuracy on standard WM tasks also showing faster RTs on the social WM task. This is also consistent with observations during testing that participants often performed the manipulation of information in WM during the probe phase, which would be reflected in their reaction time. Social WM accuracy (not analysed in this study) was not correlated with non-social WM performance (**Table S4**).

**Table S4:** Partial correlations between performance metrics on the three WM paradigms, controlling for age and sex. Significant effects are indicated in bold, and significance values are reported as *: *p* < .05, **: *p* < .01, ***: *p* < .001.

|  | **Visuospatial WM score** | **Visuospatial WM RT** | **Social WM  RT** | **Social WM accuracy** |
| --- | --- | --- | --- | --- |
| **Backwards digit score** | **.371***** | .093 | **-.143*** | -.092 |
| **Visuospatial WM score** |  | **.426***** | **-.158**** | -.081 |
| **Visuospatial WM RT** |  |  | .082 | -.003 |

**Supplementary References**

Akaike, H. (1974). A new look at the statistical model identification. *IEEE Transactions on Automatic Control*, *19*, 716–723. *doi:* 10.1109/TAC.1974.1100705

Alloway, T. P., & Alloway, R. G. (2013). Working memory across the lifespan: A cross-sectional approach. *Journal of Cognitive Psychology*, *25*(1), 84–93. doi: 10.1080/20445911.2012.748027

Barnett, J. H., Scoriels, L., & Munafò, M. R. (2008). Meta-analysis of the cognitive effects of the catechol-O-methyltransferase gene Val158/108Met polymorphism. *Biological Psychiatry*, *64*, 137–144. *doi:* 10.1016/j.biopsych.2008.01.005

Cansino, S., Hernández-Ramos, E., Estrada-Manilla, C., Torres-Trejo, F., Martínez-Galindo, J. G., Ayala-Hernández, M., et al. (2013). The decline of verbal and visuospatial working memory across the adult life span. *Age*, *35*, 2283-302. doi: 10.1007/s11357-013-9531-1

Dumontheil, I., Jensen, S. K. G., Wood, N. W., Meyer, M. L., Lieberman, M. D., & Blakemore, S.-J. (2014). Preliminary investigation of the influence of dopamine regulating genes on social working memory. *Social Neuroscience*, *9*, 437–451. *doi:* 10.1080/17470919.2014.925503

Dumontheil, I., Roggeman, C., Ziermans, T., Peyrard-Janvid, M., Matsson, H., Kere, J., & Klingberg, T. (2011). Influence of the COMT genotype on working memory and brain activity changes during development. *Biological Psychiatry*, *70*, 222–9. *doi:* 10.1016/j.biopsych.2011.02.027

Hale, S., Rose, N. S., Myerson, J., Strube, M. J., Sommers, M., Tye-Murray, N., & Spehar, B. (2011). The structure of working memory abilities across the adult life span. *Psychology and aging*, *26*, 92-110. *doi:* 10.1037/a0021483

Howell, D. C. (1997). *Statistical Methods for Psychology* (4th ed.). London: Duxbury Press.

Schwartz, G. (1978). Estimating the dimension of a model. *The Annals of Statistics*, *6*, 461–464. *doi:* 10.1214/aos/1176344136

1. While inverse and linear models were not nested and therefore could not be compared through F-tests, hierarchical regressions indicated that the quadratic age term explained a significant amount of additional variance compared to the linear model (backwards digit score: *R*^2^ = 22.6%, Δ*R*^2^ = 5.3%, *F*(1, 329) = 22.6, *p* < .001; visuospatial WM score: *R*^2^ = 7.6%, Δ*R*^2^ = 3.9%, *F*(1, 329) = 13.9, *p* < .001; social WM mean RT: *R*^2^ = 14.3%, Δ*R*^2^ = 9.5%, *F*(1, 307) = 33.9, *p* < .001). [↑](#footnote-ref-1)
